# Modified retrograde ESIN for diametaphyseal distal radius fractures in children: a retrospective cohort study

**DOI:** 10.1186/s12891-026-09952-1

**Published:** 2026-05-16

**Authors:** Raffael Cintean, Carlos Pankratz, Konrad Schütze, Florian Gebhard, Alexander Eickhoff

**Affiliations:** 1 Department of Trauma-, Hand-, and Reconstructive Surgery, Ulm University, Albert- Einstein-Allee 23, Ulm, 89081 Germany; 2 Orthopädie und Unfallchirurgie, Gesamtklinikum Mittelrhein, Koblenzer Straße 115-155, Koblenz, 56073 Germany

**Keywords:** ESIN, Diametaphyseal radius fractures, Pediatric trauma, Intramedullary nailing

## Abstract

**Background:**

Diametaphyseal distal radius fractures in children represent a therapeutic challenge because conventional elastic stable intramedullary nailing (ESIN) may provide insufficient distal fragment control, while alternative fixation methods are more invasive. This study evaluates a modified retrograde ESIN technique with a 90° distal bend (“crutch-bend technique”) designed to improve distal fragment support and alignment.

**Methods:**

In this retrospective single-center study, 43 pediatric patients with diametaphyseal distal radius fractures treated between 2022 and 2025 were analyzed. Patients received either standard ESIN (*n* = 21) or modified crutch-bend ESIN (*n* = 22). Coronal and sagittal angulation were measured on standard anteroposterior and lateral radiographs. Revision surgery, fracture healing, and clinical status at follow-up were recorded.

**Results:**

Postoperative coronal angulation was significantly lower in the modified ESIN group (3.8 ± 1.2°) compared with the standard ESIN group (8.1 ± 1.5°), corresponding to a mean difference of 4.3° (95% CI 3.5–5.1; *p* < 0.0001). Sagittal angulation was also reduced (2.1 ± 1.1° vs. 3.2 ± 0.9°; mean difference 1.1°, 95% CI 0.5–1.7; *p* = 0.0007). Revision surgery for secondary displacement occurred in two patients (9.5%) after standard ESIN and in none after the modified technique (*p* = 0.233). All fractures achieved radiographic union within approximately four weeks, and no clinically documented functional limitations were reported at follow-up.

**Conclusions:**

The crutch-bend modification of retrograde ESIN was associated with improved postoperative radiographic alignment in this retrospective cohort. Whether this translates into clinically meaningful advantages remains uncertain and requires further investigation in larger prospective studies.

## Introduction

 Fractures of the forearm are among the most common injuries in childhood, accounting for approximately 38% of all fractures in this age group, with distal fractures representing nearly two thirds of cases [1–3]. While percutaneous Kirschner wire fixation is widely accepted as the standard treatment for metaphyseal fractures, and elastic stable intramedullary nailing (ESIN) is considered the gold standard for diaphyseal fractures, fractures occurring at the diametaphyseal junction remain particularly challenging to manage [1, 4].

Radiographically, the diametaphysis represents the transitional zone between metaphysis and diaphysis, characterized by increasing cortical thickness and decreasing remodeling potential [1, 5, 6]. Consequently, fractures in this region often demonstrate substantial instability while simultaneously offering limited capacity for spontaneous correction, particularly in older children and adolescents [4, 7, 8]. As a result, standard fixation techniques frequently fail to provide sufficient control of the distal fragment in diametaphyseal fractures. Conventional ESIN in this zone may therefore result in axial malalignment or rotational instability with a risk of secondary displacement. Conversely, percutaneous Kirschner wire fixation is technically demanding and frequently requires additional immobilization, whereas plate fixation, although biomechanically stable, is associated with greater soft-tissue trauma and surgical invasiveness [9, 10].

To address these limitations, several technical modifications have been proposed, including transepiphyseal Kirschner wire fixation and customized prebending of ESIN implants to improve distal fragment control and reduce complication rates. However, no consensus has been established regarding an optimal minimally invasive stabilization strategy for diametaphyseal distal radius fractures [9, 11].

The modified retrograde ESIN technique presented in this study, referred to as the crutch-bend technique, introduces a 90° bend at the distal end of the intramedullary nail to enhance support of the distal fragment and improve axial alignment. The aim of this study was to explore whether a modified ESIN technique may improve radiographic alignment compared with standard ESIN in pediatric patients with diametaphyseal distal radius fractures.

We hypothesized that the modified technique may provide improved control of the distal fragment and thereby reduce the risk of secondary displacement.

## Materials and methods

This retrospective single-center cohort study included all pediatric patients who underwent operative treatment for diametaphyseal distal radius fractures between January 2022 and December 2025. Patients with proximal or purely diaphyseal fractures as well as those treated with alternative fixation techniques were excluded. Fractures included in the study showed varying degrees of displacement typical for diametaphyseal injuries. In total, 43 patients met the inclusion criteria. The choice of surgical technique was based on surgeon preference and experience. Based on the surgical technique applied, patients were assigned to treatment with standard elastic stable intramedullary nailing (ESIN; *n* = 21) or a modified retrograde ESIN using a distal 90° bend of the nail tip (crutch-bend technique; *n* = 22). Two surgeons consistently applied the modified ESIN technique in diametaphyseal fractures, whereas other surgeons used standard ESIN techniques.

**Fig. 1:**
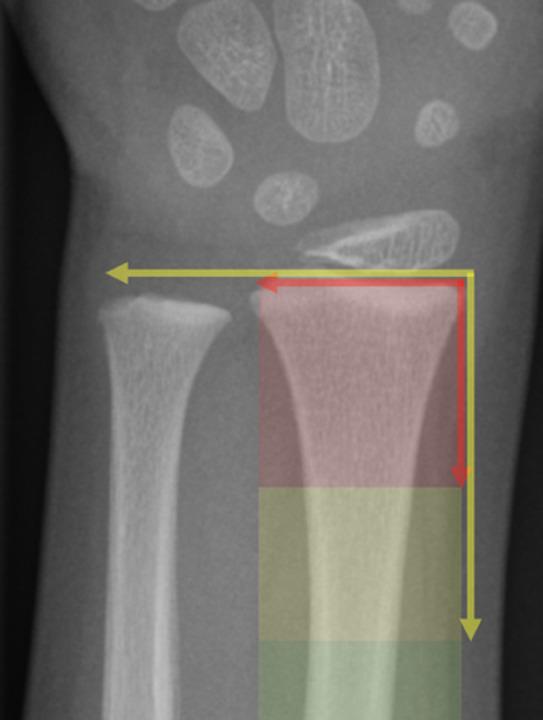
Radiographic definition of metaphyseal (red), diametaphyseal (yellow), and diaphyseal (green) regions of the distal radius based on projection of distal radial width onto the longitudinal axis

Fracture localization was determined radiographically using previously described criteria [1, 10]. The maximal transverse width of the distal radius was projected onto the longitudinal axis of the bone. The segment corresponding to this projected length defined the metaphyseal region, the immediately proximal segment of identical length the diametaphyseal region, and fractures located beyond this level were considered diaphyseal (Fig. 1).

In both groups, retrograde intramedullary nailing was performed using standard entry points at the distal radius. Nail diameter was selected according to the medullary canal size, typically corresponding to approximately two-thirds of the canal diameter. Closed reduction was performed under fluoroscopic guidance prior to nail insertion.

**Fig. 2:**
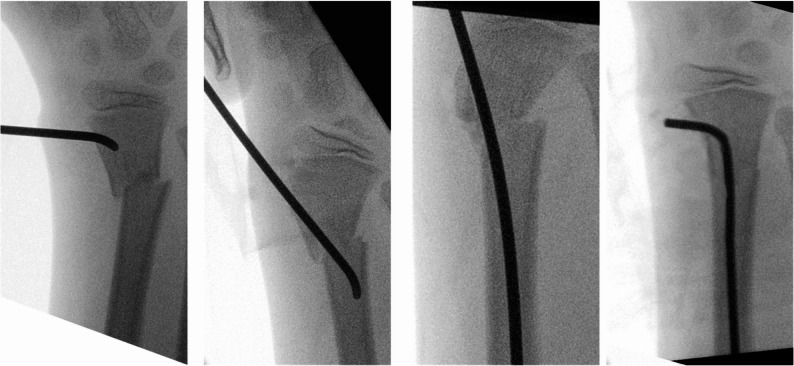
Intraoperative fluoroscopic views of a diametaphyseal distal radius fracture treated with modified retrograde ESIN using a distal 90° bend (“crutch-bend technique”)

**Fig. 3:**
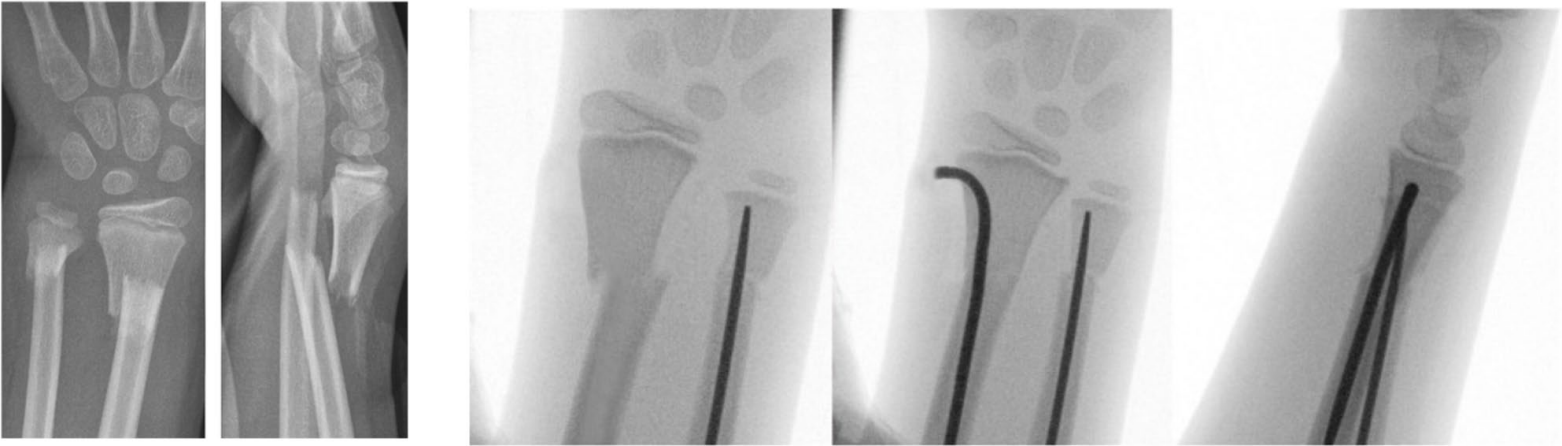
Pre- and intraoperative radiographs of a distal forearm fracture in a 9-year-old child treated with the modified retrograde ESIN technique in the radius and antegrade ESIN fixation of the ulna

In the modified technique, the nail was advanced to the distal fragment and subsequently bent to approximately 90° at its distal end to enhance engagement of the distal fragment. Reduction and implant position were continuously controlled using fluoroscopy, with particular attention to coronal and sagittal alignment (Fig. 2, 3). Final stability was assessed intraoperatively based on alignment and resistance to displacement during gentle manipulation.

Demographic data, associated injuries, and radiographic parameters were analyzed. Pre- and postoperative coronal and sagittal angulation were measured on standardized anteroposterior and lateral radiographs using the longitudinal axis of the radial shaft and the distal articular segment. Measurements were performed independently by two observers. Interobserver reliability was assessed using the intraclass correlation coefficient (ICC) based on a two-way random-effects model with absolute agreement. No routine radiographs of the contralateral side were obtained.

The primary outcome measure was postoperative coronal angulation. Secondary outcomes included sagittal angulation, the occurrence of revision surgery due to secondary displacement, fracture healing time, and clinical status at follow-up, which was recorded based on routine clinical examination and documentation, including the absence of relevant functional limitations. Revision surgery was indicated in cases of clinically relevant secondary displacement on follow-up radiographs, considering progressive angulation, fracture instability, and the expected remodeling potential based on patient age.

Continuous variables are presented as mean ± standard deviation. Postoperative angulation between groups was compared using Welch’s t-test, while revision rates were analyzed with Fisher’s exact test. A p-value below 0.05 was considered statistically significant.

## Results

A total of 43 pediatric patients with a mean age of 10.2 years (range 2–16 years) were included in the analysis. An associated distal ulna fracture was present in 26 cases. Follow-up was continued until implant removal, which was performed after radiographic fracture healing had been confirmed. Interobserver reliability was good for coronal angulation (ICC = 0.87, 95% CI 0.78–0.93) and excellent for sagittal angulation (ICC = 0.91, 95% CI 0.84–0.95). Baseline characteristics were comparable between groups, with no significant differences in age, sex, fracture characteristics, or preoperative angulation (Table 1).

**Table 1:** Baseline characteristics

Parameter	Standard ESIN (*n* = 21)	Modified ESIN (*n* = 22)	*p*-value
Age (years)	9.5 ± 6.1	11.1 ± 4.1	0.286
Sex (Male/Female, n)	15 / 6	16 / 6	0.942
Associated ulna fracture (n, %)	12 (57.1%)	14 (63.6%)	0.741
Coronal angulation (°)	20.4 ± 14.3	23.1 ± 16.4	0.425
Sagittal angulation (°)	31.6 ± 24.9	34.4 ± 26.3	0.562

Postoperative alignment differed markedly between treatment groups. In the coronal plane, residual angulation after standard ESIN measured 8.1 ± 1.5°, whereas the modified crutch-bend ESIN resulted in significantly lower angulation of 3.8 ± 1.2°, corresponding to a mean difference of 4.3° (95% confidence interval [CI] 3.5–5.1; *p* < 0.0001).

Similarly, sagittal angulation was reduced from 3.2 ± 0.9° following standard ESIN to 2.1 ± 1.1° after the modified technique, yielding a mean difference of 1.1° (95% CI 0.5–1.7; *p* = 0.0007). Maximum postoperative coronal angulation reached 19.1° in the standard group compared with 9.1° in the modified ESIN group.

Revision surgery due to secondary fracture displacement was required in two patients (9.5%) treated with standard ESIN, while no revisions occurred after the modified technique (Fisher’s exact test, *p* = 0.233, see Table 2).

**Table 2:** Postoperative alignment and revision outcomes

Parameter	Standard ESIN (*n* = 21)	Modified ESIN (*n* = 22)	Mean difference (95% CI)	*p*-value
Coronal angulation (°)	8.1 ± 1.5	3.8 ± 1.2	4.3 (3.5–5.1)	< 0.0001*
Sagittal angulation (°)	3.2 ± 0.9	2.1 ± 1.1	1.1 (0.5–1.7)	0.0007*
Revision surgery, n (%)	2 (9.5%)	0 (0%)	—	0.233†

* Welch’s test

† Fishers exact test

All fractures achieved radiographic union after approximately four weeks, and no clinically documented functional limitations were reported at follow-up.

## Discussion

The present study demonstrates that the modified retrograde ESIN technique with a distal 90° bend (“crutch-bend technique”) was associated with improved postoperative alignment in pediatric diametaphyseal distal radius fractures compared with standard ESIN. Residual angulation was reduced in both the coronal and sagittal planes, while no revision surgery due to secondary displacement was observed in the modified group.

Fractures at the diametaphyseal junction of the distal radius represent a well-recognized therapeutic challenge in pediatric trauma surgery [10, 12, 13]. The transition from metaphyseal to diaphyseal bone is associated with increasing cortical thickness and decreasing remodeling potential, particularly in older children and adolescents. Consequently, unstable fracture configurations in this region may not be adequately controlled by conventional ESIN, which relies on symmetric elastic bracing forces and sufficient distal fragment length. Previous clinical and biomechanical studies have highlighted the risk of secondary displacement and axial malalignment when ESIN is used close to the metaphysis, especially when the distal fragment-to-total radius length ratio is unfavorable [1, 4, 6, 8, 14].

The crutch-bend modification investigated in the present study addresses this limitation by creating a mechanical buttress effect within the distal fragment. The reduction in postoperative angulation observed in our cohort supports the concept that distal bending of the nail may improve control of the distal fragment in diametaphyseal fractures.

However, although the observed difference in coronal angulation was statistically significant, its clinical relevance remains uncertain. In pediatric distal radius fractures, a certain degree of residual deformity may be compensated by remodeling, particularly in younger children. In contrast, in older children and adolescents with limited remodeling potential, even moderate residual angulation may persist and potentially affect functional outcomes. Therefore, improved primary alignment may be of greater clinical importance particularly in older children and adolescents with limited remodeling potential [15–17].

Alternative fixation strategies, including percutaneous Kirschner wire stabilization or plate osteosynthesis, may improve mechanical stability but introduce relevant disadvantages. Kirschner wires often require additional immobilization and carry a risk of pin-related complications, whereas plate fixation is associated with greater surgical invasiveness, soft-tissue trauma, and implant-related morbidity [5, 9, 18, 19]. The modified ESIN technique may therefore represent a minimally invasive option to improve distal fragment control in diametaphyseal fractures.

Although no revision surgery was observed in the modified ESIN group, this finding must be interpreted cautiously. The difference in revision rate did not reach statistical significance, and the study may be underpowered to detect differences in revision rates due to the limited sample size.

Fracture healing occurred within the expected timeframe for pediatric forearm fractures, and no clinically documented functional limitations were reported at follow-up. However, functional outcomes were not assessed using validated scoring systems, which represents an important limitation of the present study. Therefore, no definitive conclusions can be drawn regarding the clinical superiority of the modified technique.

This study has several limitations. First, the retrospective design and relatively small sample size limit statistical power and increase the risk of selection bias. The relatively small sample size may also be explained by the strict radiographic definition of diametaphyseal fractures based on previously described criteria, resulting in a comparatively uncommon fracture entity. Treatment allocation was not randomized and was influenced by surgeon preference, reflecting real-world clinical decision-making but potentially introducing bias. Second, the age range of the cohort was broad (2–16 years), and remodeling potential differs substantially between younger children and adolescents approaching skeletal maturity. Third, fracture morphology was not further subclassified beyond routine clinical assessment, which may influence the applicability of the technique across different diametaphyseal fractures. In addition, the follow-up period was limited to the time of implant removal and may not be sufficient to assess long-term functional outcomes, remodeling, or late complications. Finally, although interobserver reliability of radiographic measurements was assessed, no intraobserver analysis was performed.

The present findings should therefore be interpreted as exploratory. Larger prospective studies with standardized outcome measures and longer follow-up are required to further define the clinical relevance and indications of the modified technique.

## Conclusion

The present study suggests that the crutch-bend modification of retrograde ESIN may improve postoperative alignment in pediatric diametaphyseal distal radius fractures while maintaining the minimally invasive advantages of intramedullary fixation. However, the clinical relevance of this improved alignment remains uncertain, particularly in view of the remodeling potential in younger patients. The technique may be particularly useful in diametaphyseal fractures with sufficient distal fragment length to allow stable intramedullary support. Given the retrospective design, limited sample size, and absence of standardized functional outcome assessment, the findings should be interpreted with caution. Further prospective studies with larger cohorts and long-term follow-up are required to better define the clinical indications and potential benefits of this technique.

## Data Availability

The datasets generated and analyzed during the current study are not publicly available due to institutional data protection regulations but are available from the corresponding author on reasonable request.

## Abbreviations

ESIN: Elastic stable intramedullary nailing
